# Isolated brachydactyly type E caused by a *HOXD13 *nonsense mutation: a case report

**DOI:** 10.1186/1471-2350-13-4

**Published:** 2012-01-10

**Authors:** Aleksander Jamsheer, Anna Sowińska, Leszek Kaczmarek, Anna Latos-Bieleńska

**Affiliations:** 1 Department of Medical Genetics, University of Medical Sciences in Poznan, Poland, ul. Grunwaldzka 55 paw. 15, 60-352 Poznan, Poland; 2 NZOZ Center for Medical Genetics, Poznan, Poland, ul. Grudzieniec 4, 60-601 Poznan, Poland; 3 Department of Traumatology, Orthopedics and Hand Surgery, University of Medical Sciences in Poznan, Poland, ul. 28 czerwca 1956 r. 135/147, 61-545 Poznan, Poland

**Keywords:** brachydactyly type E, BDE, isolated brachydactyly, nonsense mutation, *HOXD13*

## Abstract

**Background:**

Brachydactyly type E (BDE; MIM#113300) is characterized by shortening of the metacarpal, metatarsal, and often phalangeal bones, and predominantly affects postaxial ray(s) of the limb. BDE may occur as an isolated trait or as part of a syndrome. Isolated BDE is rare and in the majority of cases the molecular pathogenesis has so far not been resolved. Originally, the molecular cause of isolated BDE has been unravelled in 2 families and shown to result from heterozygous missense mutations in the homeodomain of the *HOXD13 *gene. Since the initial manuscript, one further *HOXD13 *mutation has been reported only in a single family manifesting isolated BDE.

**Case Presentation:**

In this paper, we report on a Polish family exhibiting isolated BDE caused by a novel nonsense heterozygous *HOXD13 *mutation. We investigated a Polish female proband and her father, both affected by isolated BDE, in whom we identified a nonsense heterozygous mutation c.820C > T(p.R274X) in the *HOXD13 *gene. So far, only two missense *HOXD13 *substitutions (p.S308C and p.I314L), localized within the homeodomain of the HOXD13 transcription factor, as well as a single nonsense mutation (p.E181X) were associated with BDE. Both missense changes were supposed to alter DNA binding affinity of the protein.

**Conclusion:**

The variant p.R274X identified in our proband is the fourth *HOXD13 *mutation, and the second truncating (nonsense) mutation, reported to result in typical isolated BDE. We refer our clinical and molecular findings to the previously described *HOXD13 *associated phenotypes and mutations.

## Background

Brachydactyly type E (BDE; MIM#113300) is characterized by shortening of the metacarpal, metatarsal, and often phalangeal bones, and predominantly affects postaxial ray(s) of the limb [1]. In most cases BDE is syndromic and occurs within the clinical spectrum of Turner syndrome, Albright hereditary osteodystrophy (AHO; MIM#103580) or 2q37 deletion [1]. Isolated BDE is rare and in the majority of cases has unknown genetic background. Originally, the molecular cause of isolated BDE has been unravelled in 2 families and shown to result from heterozygous missense mutations in the homeodomain of the *HOXD13 *gene [2]. Some patients from these families presented not only with BDE, but also with overlapping features of brachydactyly type D (BDD), defined as shortening and broadening of the thumbs [2].

**Figure 1:**
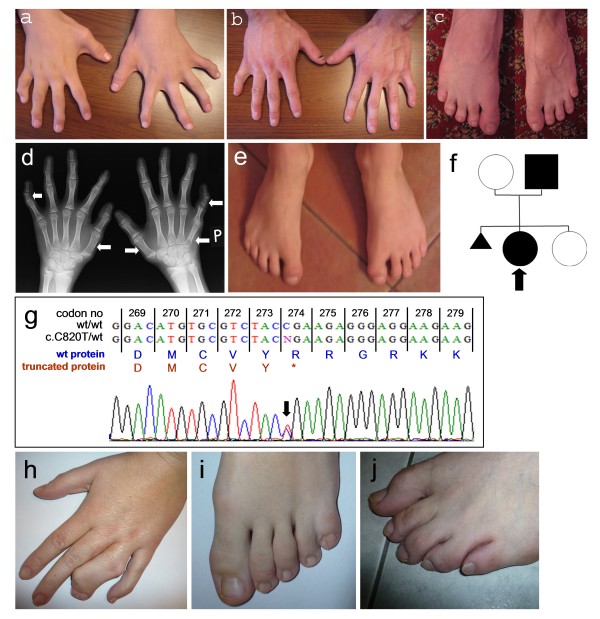
**A-Brachydactyly type E (BDE) in the proband characterized by shortened V^th ^fingers, B, C-Clinical picture of BDE (shortened V^th ^fingers and toes) in the proband's father, D-X-ray of the proband's hands showing shortening of the V^th ^right metacarpal, bilateral shortening and broadening of the I^st ^metacarpals, and bilateral shortening of the middle phalanges of V^th ^fingers (skeletal abnormalities are indicated by the white arrows), E-Feet of the proband showing broad halluces and no clinically evident BDE, F-Pedigree of the presented BDE family (proband is indicated by an arrow), G-Chromatogram picture showing the c.820C > T(p.R274X) *HOXD13 *mutation**. Wild-type HOXD13 protein sequence is presented in blue, whereas truncated protein variant is shown in red. **H **- Hand malformation typical for synpolydactyly (SPD) spectrum (syndactyly of fingers 3/4 with insertional polydactyly within the syndactylous web), **I **- Typical SPD foot malformation characterized by syndactyly of 4/5 toes, **J **- External rotation of 5^th ^toe often seen in SPD patients (SPD in patients shown in pictures H, I, J results from insertions of 7 Ala in the polyalanine tract).

Missense variants affecting other residues of the C-terminal HOXD13 homeodomain may also give rise to different and more severe limb phenotypes such as synpolydactyly (SPD; MIM#186000) or syndactyly type 5 (MIM#186300), whereas expansion or contraction of the N-terminal HOXD13 polyalanine tract usually results in SPD [3-6]. Most patients affected by SPD show fusion of third and fourth fingers and fourth and fifth toes, accompanied with incomplete or complete insertional polydactyly within the syndactylous web. Affected individuals may also have external rotation of fifth toes, clinodactyly, and camptodactyly (Figure 1H, I &1J present a family with classical SPD caused by insertion of 7 alanines within the HOXD13 polyalanine tract). The clinical presentation of SPD is highly heterogeneous and varies within the affected individual (asymmetrical expressivity) or in the family. Severe manifestation and incomplete penetrance may occur within the same pedigree [4,5].

**Figure 2:**
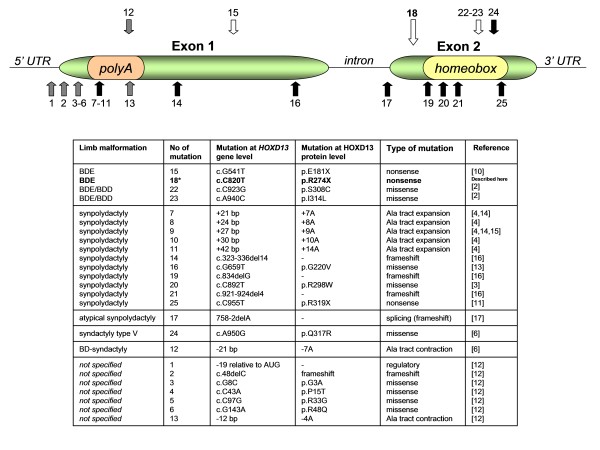
**Schematic presentation of the *HOXD13 *gene structure and overview of all annotated *HOXD13 *mutations identified in patients affected by various limb malformations**. Arrows indicate approximate positions of *HOXD13 *mutations. Numbers below or above each arrow refer to specific mutations and correspond to the numbers provided in the table below. Different designations have been used to discriminate between types of mutations: arrows above the exons indicate mutations causing BDE, BD-syndactyly, and syndactyly type V (white, grey, and black arrows respectively); arrows below the exons show mutations resulting in SPD or unspecified limb phenotype (black and grey arrows respectively). Mutation p.R274X identified in our proband (number 18) has been bolded and highlighted with large arrow.

*HOXD13 *is a member of a large family of developmental homeobox transcription factor genes. Human, as well as other vertebrate genomes contain 39 *HOX *genes organized into four clusters (*HOXA, HOXB, HOXC*, and *HOXD*). *HOX *genes are involved in body plan formation and embryonic development of many internal organs, such as central nervous system, gastrointestinal and genitourinary tract. They also play a critical role in limb development by influencing limb patterning along proximodistal and anteroposterior axes. In general, the position of the gene in each cluster corresponds to its spatio-temporal expression during limb development in an order from 3' to 5' end. Thus, homologues located at the 3' end of the cluster are expressed earlier in development and in more proximal and anterior structures, whereas those located at the 5' end are expressed later and in more posterior and distal embryonic regions [7,8]. The *HOXD13 *gene consists of two exons and encodes a protein built of 335 amino acids. Exon 1 contains an N-terminal tract comprised of 15 polyalanine residues in wild-type protein. Exon 2 carries a sequence for a highly conserved C-terminal DNA binding domain, known as homeodomain, through which HOXD13 interacts with consensus DNA sequence. A schematic view of the protein structure and a summary of annotated mutations are given in Figure 2.

Recently, Klopocki et al. [9] described causative alterations (microdeletion and point mutations) in the *PTHLH *gene in five unrelated families affected by BDE and short stature. Since the initial paper of Johnson et al. from 2003 [2], *HOXD13 *mutation has been reported only in a single family manifesting isolated BDE [10]. In addition, only 8 out of 24 annotated *HOXD13 *mutations resulted in a premature termination of the protein synthesis [10-17]; see Figure 2). Thus, genotype-phenotype correlation for truncating HOXD13 variants remains poorly known.

In this paper, we relate on a Polish family exhibiting isolated BDE caused by a nonsense heterozygous *HOXD13 *mutation and refer our clinical and molecular finding to previously described *HOXD13 *associated phenotypes and mutations.

### Case presentation

We investigated a 10 year old female proband and her 28 year old father of Polish origin, both affected by isolated BDE (a family tree is shown in Figure 1F). Skeletal manifestations of the proband (Figure 1A, D &1E) comprised shortening of the V^th ^right metacarpal without shortening of IV^th ^metacarpal bones, bilateral shortening and broadening of the I^st ^metacarpals, bilateral shortened, trapezoid middle phalanges of V^th ^fingers resulting in rather severe clinodactyly, as well as contractures and deviations of the fingers II-IV. Upon clinical examination feet of the proband showed broad halluces and no clinically recognizable BDE phenotype. Unfortunately, since no X-ray was available, we were unable to rule out all possible skeletal foot abnormalities. The proband's father (Figure 1B &1C) manifested finger contractures, shortening of the V^th ^fingers and toes most probably due to shortened V^th ^metacarpals and metatarsal, shortened finger nails of the V^th ^fingers most likely resulting from hypoplastic distal phalanges, as well as short thumbs. Unfortunately, the patient decided not to undergo X-ray examination, thus we were unable to delineate skeletal findings in more detail. Both patients had normal stature and normal psychomotor development.

Genomic DNA was extracted from peripheral blood leukocytes according to salting-out method [18]. The entire coding sequence of the *HOXD13 *gene comprised of two exons and the flanking intronic regions (GenBank NM_000523.3) were amplified in PCR reactions and directly sequenced using dye-terminator chemistry (kit v.3, ABI 3130XL). Sequences of the primers used for amplification and sequencing PCR reactions are given in table 1. PCR conditions used for *HOXD13 *amplification where as follows: 40 cycles, denaturation in 95°C (30'), annealing (30') with temperature starting from 63°C, decreasing to 55°C (touchdown PCR -0.2°C per cycle), and elongation in 72°C (45'). Multiplex ligation-dependent probe amplification (MLPA) for both exons of the *HOXD13 *was performed by means of P179 commercial kit according to the manufacturer's protocol (MRC Holland).

**Table 1:** Sequences of the primers used for *HOXD13 *gene amplification and sequencing.

Exon name (fragment)	Forward primer sequence 5'- 3'	Reverse primer sequence 5'- 3'	Product size (bp)
HOXD13_e1(a)	TATAAACGTCCCGCGATGAG	ATTCTGCTGTAAGCCCACGC	644

HOXD13_e1(b)	CAAAGAGTGCCCAGCACC	TAACCCTGGTCACGTGTGG	599

HOXD13_e2	AAAATTTCCTGCACCCCTG	CACAAAATTTGCCACCATTG	491

The proband and her father carried a nonsense heterozygous mutation c.820C > T(p.R274X) in the *HOXD13 *gene (Figure 1G). Presence of this mutation was excluded in 208 ethnically matched control chromosomes. MLPA ruled out intragenic copy number changes within the *HOXD13 *in both proband and her father (i.e. deletion/duplication).

## Discussion

So far, only a few *HOXD13 *mutations are known to result in an isolated brachydactyly phenotype [2,10]. Originally, two missense mutations (p.S308C and p.I314L), localized within the homeodomain of the HOXD13 transcription factor were associated with overlapping features of BDE and BDD [2]. Both changes were supposed to alter DNA binding affinity of the protein [2]. Additionally, one nonsense variant p.E181X was described by Brison et al. [10] in a Belgian family presenting with isolated BDE, but neither detailed clinical description nor pictures were provided for this case. The variant p.R274X identified in our proband and her father is the fourth *HOXD13 *mutation, and the second truncating (nonsense) mutation reported to result in typical isolated BDE/BDD. Noteworthy, clinical manifestation of BDE in our patients shared significant similarities with the features reported by Johnson et al. [2] in the two original families. In addition to metacarpal brachydactyly (BDE), both ours and Johnson's patients had clinodactyly, deviations, and contractions of fingers, short thumbs with apparently hypoplastic distal phalanges, and hypoplastic middle and distal phalanges, especially of the little fingers. Unlike Johnson's families, none of our patients showed syndactyly of 3/4, ring finger duplication or long distal digit phalanges. Moreover, metacarpal brachydactyly in our case was confined solely to V^th ^digits, whereas in other BDE patients also affected other rays [1,2].

The mutant HOXD13 protein (p.R274X) synthesized in our proband is predicted to lack the entire homeodomain and hence cannot bind to DNA consensus. Likewise, the same pathogenic mechanism most probably exists in the case of p.E181X variant which also results in isolated BDE (see Figure 2). On the other hand, the most terminal of all known truncating *HOXD13 *mutation (p.R319X), which is localized at the very end of the homeodomain sequence, produces SPD. Out of truncating *HOXD13 *alterations, four frameshift and one splicing mutation have been additionally described in the literature. Four of them caused a phenotype referred to as "SPD with foot anomalies" (MIM#186000), in which classic SPD was accompanied by supernumerary digit between I^st^-II^nd ^and often IV^th^-V^th ^metatarsals [16,17]. The most plausible mechanism through which all truncating mutations exert their pathogenic effect is functional haploinsufficiency of *HOXD13*. Conversely, missense substitutions affecting different residues of homeodomain alter rather than abolish DNA binding ability of the mutant protein. Hence, mixed both gain- and loss-of-function mechanisms have been postulated to account for different limb phenotypes associated with these mutations [2,6]. Despite different mutational mechanisms of p.I314L and p.S308C substitutions in reference to our mutation (p.R274X), there has been a substantial overlap in the clinical phenotype. More severe presentation associated with missense mutations can be thus explained by their dominant-negative effect.

Another example of a homeotic gene known from its pleiotropic pathogenic effect is a *HOXD13 *paralogue belonging to a HOXA cluster-*HOXA13*. Mutations in this gene may give rise to different phenotypes, depending on their type and intragenic location. While nonsense truncating mutations N-terminal to or within the homeodomain, as well as expansions of polyalanine tract cause Hand-foot-genital syndrome (HFGS also known as Hand-foot-uterus syndrome; HFUS; MIM#140000), missense mutation within the homeodomain has been associated with Guttmacher syndrome (MIM #176305), a phenotype in which HFGS features are accompanied with postaxial polydactyly of the hands and uniphalangeal second toes [19,20].

In the developmental context, *Hoxd13 *has been shown to suppress chondrogenesis in the interdigital space, thus being responsible for proper digit formation. A loss-of-function mutation in mouse *Hoxd13 *results in down-regulation of *Raldh2 *and thereby in low tissue concentration of retinoic acid. This induces expression of *Sox9 *in the interdigital mesenchyme, the formation of interdigital condensation, and consequently, polydactyly [21]. Conversely, missense mutations of human *HOXD13 *localized in the homeodomain exert their pathogenicity via gain-of-function effect that possibly reduces level of *SOX9 *expression. Of note, *Sox9 *is also a target molecule of *Pthlh *(*Pthrp*) signaling in prehypertrophic chondrocytes in the growth plate. *Pthlh *increases transcriptional activity of *Sox9*, helps to maintain chondrocyte phenotype of the cells in the prehypertrophic zone, and inhibits their differentiation to hypertrophic chondrocytes [22]. Hence, haploinsufficiency of human *PTHLH *in BDE patients may at least partly result in reduced transcriptional activity of *SOX9*, which fails to maintain the chondrocyte phenotype in prehypertrophic zone and, in turn, promotes their maturation to hypertrophic chondrocytes.

## Conclusions

Isolated BDE can be caused by either *HOXD13 *nonsense mutations or missense substitutions within the homeodomain of the HOXD13 transcription factor. *HOXD13 *is a pleiotropic gene associated with various limb malformations. Most of the mutations occurring within the gene produce SPD, and not BDE.

## Consent

Written informed consent was obtained from the patient for publication of this case report and any accompanying images. A copy of the written consent is available for review by the Editor-in-Chief of this journal.

## Competing interests

The authors declare that they have no competing interests.

## Authors' contributions

AJ-consulted the family, conceived the manuscript; AS-performed molecular testing of the patients and controls; LK-consulted the family of interest; ALB-critically revised the manuscript. All authors read and approved the final manuscript.

## Pre-publication history

The pre-publication history for this paper can be accessed here:

http://www.biomedcentral.com/1471-2350/13/4/prepub

## References

[B1] Schwabe GC Mundlos S Genetics of congenital hand anomalies Handchir Mikrochir Plast Chir 2004 36 2-3 85 97 15162306 10.1055/s-2004-817884

[B2] Johnson D Kan S Oldridge M Trembath RC Roche P Esnouf RM Giele H Wilkie AOM Missense mutations in the homeodomain of HOXD13 are associated with brachydactyly types D and E Am J Hum Genet 2003 72 984 997 10.1086/374721 12649808 PMC1180360

[B3] Debeer P Bacchelli C Scambler PJ De Smet L Fryns JP Goodman FR Severe digital abnormalities in a patient heterozygous for both a novel missense mutation in HOXD13 and a polyalanine tract expansion in HOXA13 J Med Genet 2002 39 852 856 10.1136/jmg.39.11.852 12414828 PMC1735011

[B4] Muragaki Y Mundlos S Upton J Olsen BR Altered growth and branching patterns in synpolydactyly caused by mutations in HOXD13 Science 1996 272 5261 548 551 10.1126/science.272.5261.548 8614804

[B5] Goodman FR Mundlos S Muragaki Y Donnai D Giovannucci-Uzielli ML Lapi E Majewski F McGaughran J McKeown C Reardon W Upton J Winter RM Olsen BR Scambler PJ Synpolydactyly phenotypes correlate with size of expansions in HOXD13 polyalanine tract Proc Natl Acad Sci 1997 94 14 7458 7463 10.1073/pnas.94.14.7458 9207113 PMC23843

[B6] Zhao X Sun M Zhao J Leyva JA Zhu H Yang W Zeng X Ao Y Liu Q Liu G Lo WHY Jabs EW Amzel LM Shan X Zhang X Mutations in HOXD13 underlie syndactyly type V and a novel brachydactyly-syndactyly syndrome Am J Hum Genet 2007 80 2 361 371 10.1086/511387 17236141 PMC1785357

[B7] Krumlauf R Hox genes in vertebrate development Cell 1994 78 191 201 10.1016/0092-8674(94)90290-9 7913880

[B8] Zakany J Duboule D The role of Hox genes during vertebrate limb development Curr Opin Genet Dev 2007 17 4 359 66 10.1016/j.gde.2007.05.011 17644373

[B9] Klopocki E Hennig BP Dathe K Koll R de Ravel T Baten E Blom E Gillerot Y Weigel JF Kruger G Hiort O Seemann P Mundlos S Deletion and point mutations of PTHLH cause brachydactyly type E Am J Hum Genet 2010 86 3 434 439 10.1016/j.ajhg.2010.01.023 20170896 PMC2833367

[B10] Brison N Tylzanowski P Debeer P Limb skeletal malformations-What the HOX is going on? Eur J Med Genet 2011 doi:10.1016/j.ejmg.2011.06.003 10.1016/j.ejmg.2011.06.003 21782042

[B11] Furniss D Kan SH Taylor IB Johnson D Critchley PS Giele HP Wilkie AO Genetic screening of 202 individuals with congenital limb malformations and requiring reconstructive surgery J Med Genet 2009 46 11 730 735 10.1136/jmg.2009.066027 19429598 PMC2764122

[B12] Nakano K Sakai N Yamazaki Y Watanabe H Yamada N Sezaki K Susami T Tokunaga K Takato T Uchinuma E Novel mutations of the HOXD13 gene in hand and foot malformations Int Surg 2007 92 5 287 295 18399101

[B13] Fantini S Vaccari G Brison N Debeer P Tylzanowski P Zappavigna V A G220V substitution within the N-terminal transcription regulating domain of HOXD13 causes a variant synpolydactyly phenotype Hum Mol Genet 2009 18 5 847 860 19060004 10.1093/hmg/ddn410

[B14] Wajid M Ishii Y Kurban M Dua-Awereh MB Shimomura Y Christiano AM Polyalanine repeat expansion mutations in the HOXD13 gene in Pakistani families with synpolydactyly Clin Genet 2009 76 3 300 302 10.1111/j.1399-0004.2009.01213.x 19686284

[B15] Gong L Wang B Wang J Yu H Ma X Yang J Polyalanine repeat expansion mutation of the HOXD13 gene in a Chinese family with unusual clinical manifestations of synpolydactyly Eur J Med Genet 2011 54 2 108 111 10.1016/j.ejmg.2010.10.007 20974300

[B16] Goodman FR Giovannucci-Uzielli ML Hall C Reardon W Winter R Scambler P Deletions in HOXD13 segregate with an identical, novel foot malformation in two unrelated families Am J Hum Genet 1998 63 4 992 1000 10.1086/302070 9758628 PMC1377502

[B17] Kan S Johnson D Giele H Wilkie AOM An acceptor splice site mutation in HOXD13 results in variable hand, but consistent foot malformations Am J Med Genet 2003 121A 69 74 10.1002/ajmg.a.20103 12900906

[B18] Lahiri DK Bye S Nurnberger JI Hodes ME Crisp M A non-organic and non-enzymatic extraction method gives higher yields of genomic DNA from whole-blood samples than do nine other methods tested J Biochem Biophys Methods 1992 25 4 193 205 10.1016/0165-022X(92)90014-2 1494032

[B19] Goodman FR Bacchelli C Brady AF Brueton LA Fryns JP Mortlock DP Innis JW Holmes LB Donnenfeld AE Feingold M Beemer FA Hennekam RC Scambler PJ Novel HOXA13 mutations and the phenotypic spectrum of hand-foot-genital syndrome Am J Hum Genet 2000 67 1 197 202 10.1086/302961 10839976 PMC1287077

[B20] Innis JW Goodman FR Bacchelli C Williams TM Mortlock DP Sateesh P Scambler PJ McKinnon W Guttmacher AE A HOXA13 allele with a missense mutation in the homeobox and a dinucleotide deletion in the promoter underlies Guttmacher syndrome Hum Mutat 2002 19 5 573 574 11968094 10.1002/humu.9036

[B21] Kuss P Villavicencio-Lorini P Witte F Klose J Albrecht AN Seemann P Hecht J Mundlos S Mutant Hoxd13 induces extra digits in a mouse model of synpolydactyly directly and by decreasing retinoic acid synthesis J Clin Invest 2009 19 1 146 156 10.1172/JCI36851 PMC2613457 19075394

[B22] Huang W Chung UI Kronenberg HM de Crombrugghe B The chondrogenic transcription factor Sox9 is a target of signaling by the parathyroid hormone-related peptide in the growth plate of endochondral bones Proc Natl Acad Sci 2001 98 1 160 165 10.1073/pnas.011393998 11120880 PMC14561

